# LATS kinase activity and tumor suppressor function are regulated by a second autophosphorylation site

**DOI:** 10.1016/j.jbc.2026.113177

**Published:** 2026-05-20

**Authors:** Ruxin Jin, Zhenxing Zhong, Rui Zhu, Anlan Zhang, Jian Li, Fa-Xing Yu, Yu Wang

**Affiliations:** Institute of Pediatrics, Children's Hospital of Fudan University, Qidong-Fudan Innovative Institution of Medical Sciences, and Institutes of Biomedical Sciences, Shanghai Medical College, Fudan University, Shanghai, China

**Keywords:** activation loop, autophosphorylation, canonical LATS1/2 substrate site, Hippo signaling pathway, hydrophobic motif, kinase activity, LATS1/2, YAP/TAZ

## Abstract

The Hippo signaling pathway regulates cell proliferation, differentiation, and survival. LATS kinases (LATS1 and LATS2) are central kinases in this pathway, activated by MST/MAP4Ks through phosphorylation at the hydrophobic motif, which primes subsequent autophosphorylation at the activation loop. Here, we identify a conserved autophosphorylation site (Ser872 in LATS1 and Ser835 in LATS2) within a canonical HXRXXS motif of the kinase domain, designated as the canonical LATS1/2 substrate site. Phosphorylation at the canonical LATS1/2 substrate site is required for the full activation of LATS kinases, as substitution of this serine with alanine significantly impairs YAP phosphorylation, thereby enhancing the oncogenic activity of YAP, a key downstream effector of LATS kinases. These results provide new mechanistic insights into the regulation of LATS kinase activity and the biological function of the Hippo pathway.

The Hippo signaling pathway is a highly conserved growth control mechanism in multicellular organisms (1). By modulating cell proliferation, differentiation, and survival, the Hippo pathway plays a critical role in maintaining organ size, homeostasis, regeneration, and tumorigenesis (2). The pathway is typically depicted as a kinase cascade in which Ste20-like kinases (MST1/2, Hippo kinases) and mitogen-activated protein kinase kinase kinase kinases (MAP4K1-7, Hippo-like kinases) phosphorylate and activate the downstream large tumor suppressor kinases (LATS1/2, LATS kinases). Activated LATS kinases then phosphorylate the transcriptional cofactors Yes-associated protein (YAP) and transcriptional coactivator with PDZ-binding motif (TAZ; also known as WWTR1), thereby preventing their nuclear localization and TEAD-mediated transcriptional activation (3, 4, 5). In addition, the Hippo pathway is regulated by several scaffold proteins, including Salvador homolog 1 (SAV1), Mps one binder kinase activator 1 (MOB1A/B), neurofibromin 2 (NF2, also known as Merlin), and WW and C2 domain-containing proteins (WWC1-3). These proteins act as adaptors or modulators that coordinate multiple kinases to promote efficient signal transduction (6, 7).

LATS kinases were first identified in *Drosophila* as *Warts* (*wts*), whose inactivation leads to massive tissue overgrowth in multiple organs (8). This function is evolutionarily conserved in mammals; for example, deletion of *Lats1* or *Lats2* in mice results in tumor formation, including melanoma and peripheral nerve sheath tumors (9, 10). Furthermore, the expression of *LATS1* or *LATS2* is frequently downregulated in various human cancers, including breast, gastric, ovarian, and non-small cell lung cancers (11, 12, 13, 14, 15, 16). Collectively, these findings emphasize the crucial tumor-suppressive roles of LATS kinases.

LATS1 and LATS2 are members of the AGC kinase family and share high sequence conservation, with about 52% overall similarity and 85% homology within the kinase domain (17). Activation of LATS kinases follows a canonical two-step phosphorylation mechanism. First, the C-terminal hydrophobic motif (HM) is phosphorylated by upstream MST1/2 or MAP4K1–7 kinases, which primes LATS kinases for subsequent autophosphorylation at the activation loop (AL)—a modification essential for catalytic activation (18). Binding of MOB1A/B facilitates the phosphorylation events and stabilizes the active conformation of LATS kinases (19, 20).

Despite these insights, the regulatory landscape of LATS kinase activation remains incompletely understood. In particular, whether LATS kinases harbor additional autophosphorylation sites that fine-tune their catalytic potential remains unknown. In this study, we identify a previously unrecognized phosphorylation site within the kinase domain that undergoes autophosphorylation in response to upstream cues and modulates LATS catalytic activity, thereby shaping Hippo pathway output and physiological function.

## Results

### Identification and regulation of the CLS site

**Figure 1:**
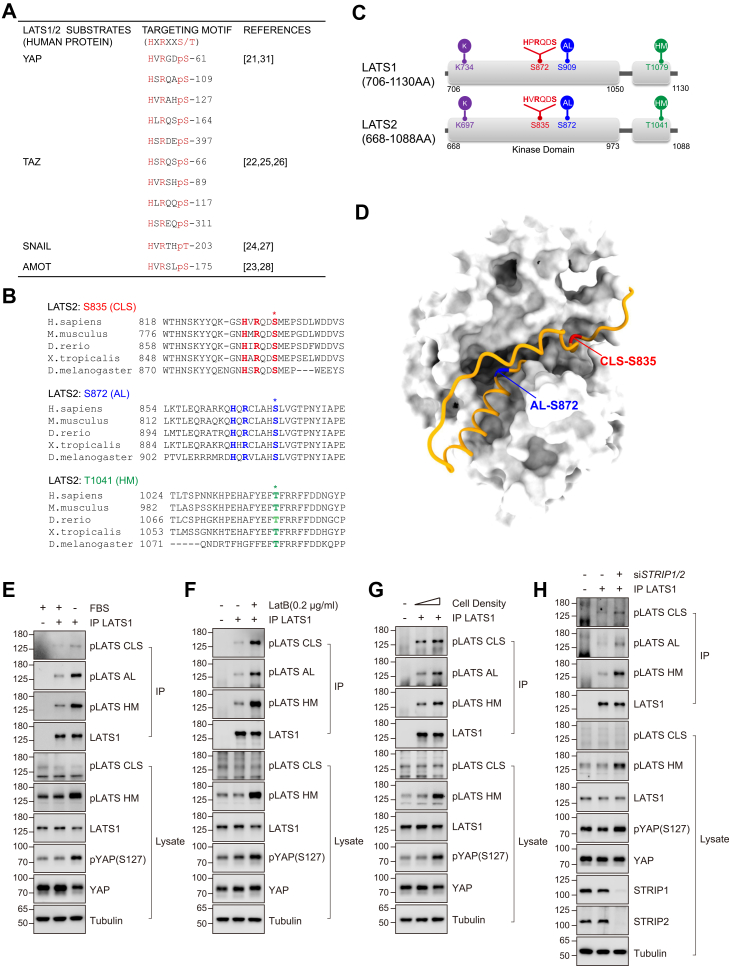
**Identification and regulation of the CLS site in LATS kinases.***A*, summary of LATS kinase substrates containing the HXRXXS/T consensus motif. H: histidine; X: any amino acid; R: arginine; S: serine; T: threonine. The key amino acid residues (H, R, and S/T) are highlighted in *red*. *B*, sequence alignment showing that the HXRXXS motif is highly conserved among LATS2 orthologs. The activation loop (AL) and hydrophobic motif (HM) sites are also evolutionarily conserved. *C*, schematic diagram showing the C-terminal kinase domains of LATS1 (706–1130 aa) and LATS2 (668–1088 aa). Key phosphorylation sites are highlighted in different colors. *D*, the structural model of the AlphaFold3-predicted C-terminal kinase domain (668–1052 aa) of human LATS2 is displayed as surface. The loop containing S835 and S872 is highlighted in *orange*, the amino acids S835 and S872 are colored *red* and *blue*, respectively. The figure was generated using ChimeraX. *E*, phosphorylation of endogenous LATS1 at the CLS site is increased upon serum deprivation. HEK293A cells were serum-starved for 0 or 12 h. Endogenous LATS1 was immunoprecipitated (IP) and analyzed by immunoblotting for phosphorylation at the CLS, AL, and HM sites. *F*-*G*, phosphorylation of LATS1 at the CLS site is induced by actin cytoskeleton disruption (*F*) or high cell density (*G*). HEK293A cells were treated with 0.2 μg/ml LatB for 30 min or cultured at increasing cell densities. Endogenous LATS1 was immunoprecipitated and analyzed. *H*, phosphorylation of LATS1 at the CLS site is enhanced upon *STRIP1/2* deletion. HEK293A cells were transfected with siRNAs targeting *STRIP1/2*, and endogenous LATS1 was immunoprecipitated and analyzed by immunoblotting. Increased YAP S127 phosphorylation indicates activation of LATS kinases.

Substrates of LATS kinases usually contain a consensus HXRXXS/T motif, where H is histidine, X is any amino acid, R is arginine, and S (serine) or T (threonine) is directly phosphorylated by LATS kinases (Fig. 1*A*) (21, 22, 23, 24, 25, 26, 27, 28). Interestingly, we identified a conserved serine residue (Ser872 in LATS1 and Ser835 in LATS2) embedded in a canonical HXRXXS motif within the kinase domain of LATS kinases. This serine is distinct from the known AL (Ser909 in LATS1 and Ser872 in LATS2) and HM (Thr1079 in LATS1 and Thr1041 in LATS2) phosphorylation sites (Fig. 1, *A*–*C*). Given the perfect match to the substrate consensus, we designated this residue as the canonical LATS1/2 substrate (CLS) site. We ectopically expressed and purified WT human LATS2 and performed phosphopeptide mapping by mass spectrometry. The analysis indicated that, in addition to the AL (Ser872) and HM (Thr1041) sites, the CLS residue (Ser835) was also phosphorylated (Fig. S1). Notably, these three phosphorylation sites are present in LATS1 and LATS2 and are evolutionarily conserved across species (Figs. 1*B* and S2*A*). Based on the predicted structure of LATS2 generated by AlphaFold, both CLS and AL sites are located in the same loop near the activation center, suggesting a potential role for CLS phosphorylation in regulating LATS kinase activity (Fig. 1*D*).

To further characterize the CLS phosphorylation, we generated a phospho-specific antibody targeting this site (hereafter referred to as the pCLS antibody). The pCLS antibody recognized immunoprecipitated LATS1 (endogenous) and LATS2 (ectopically expressed) but failed to do so in the presence of λ-phosphatase, which removes phosphate groups from proteins (Fig. S2, *B*–*D*). Moreover, the pCLS antibody was unable to recognize the LATS2 CLS-A (S835A, serine-to-alanine) mutant, which mimics the dephosphorylated state of the CLS site (Fig. S2*E*). To further confirm antibody specificity and exclude cross-reactivity with the AL site, we synthesized phosphorylated CLS (pCLS), nonphosphorylated CLS (non-pCLS), and phosphorylated AL (pAL) peptides. Dot blot analysis showed that the pCLS antibody bound strongly to the pCLS peptide in a concentration-dependent manner, with low affinity for the non-pCLS and pAL peptides (Fig. S2*F*). Consistently, peptide competition assays demonstrated that preincubation with the pCLS peptide abolished antibody binding to LATS2, whereas the non-pCLS and pAL peptides had a marginal effect (Fig. S2, *G* and *H*). Together, these data indicate that the pCLS antibody is specific for LATS1/2 phosphorylated at the CLS site.

The Hippo signaling pathway integrates various cues to regulate cell proliferation, death, differentiation, and organ size (3). As a central signaling node of the Hippo pathway, LATS kinases are regulated by upstream signals, including cell polarity, mechanical cues, cell density, diffusible factors, and stress signals (3, 29, 30, 31, 32, 33). We have previously shown that serum starvation can activate the Hippo signaling pathway, leading to the phosphorylation and activation of LATS kinases (4). We purified endogenous LATS1 from serum-starved cells and examined phosphorylation at multiple regulatory sites. The phosphorylation at the CLS site, similar to the AL and HM sites, increased upon serum starvation (Fig. 1*E*). Consistent results were observed when cells were treated with Latrunculin B (an agent that depolymerizes filamentous actin) or cultured at high cell density (Fig. 1, *F* and *G*) (31, 34, 35, 36). Additionally, the striatin-interacting phosphatase and kinase (STRIPAK) complex is a key negative regulator of the Hippo pathway (37, 38, 39, 40). Knockdown of striatin-interacting protein 1 and 2 (*STRIP1/2*, key components of the STRIPAK complex) activated the Hippo pathway and enhanced LATS1 CLS phosphorylation (Fig. 1*H*). The activity of LATS1/2 was induced under these treatments, as shown by the increased YAP phosphorylation at the S127 site (Fig. 1, *E*–*H*). Together, these results establish the CLS of LATS kinases as a phosphorylation site modulated by upstream signals of the Hippo pathway.

### CLS functions as an autophosphorylation site of the LATS kinases

**Figure 2:**
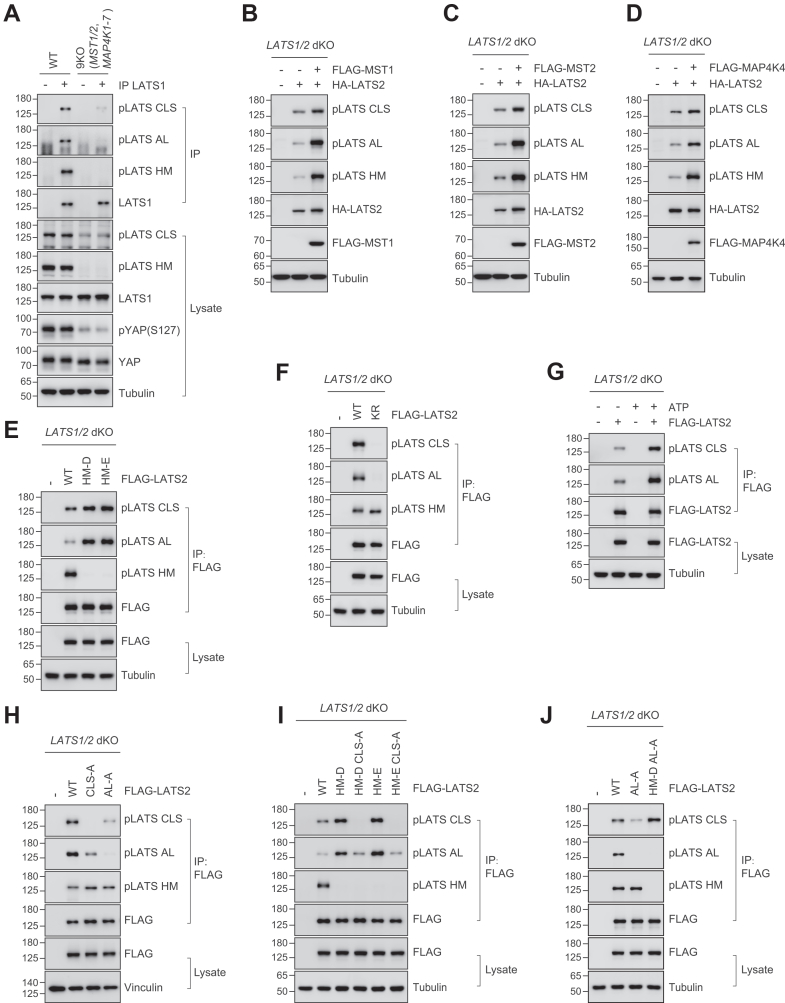
**Autophosphorylation of LATS2 at the CLS site.***A*, phosphorylation of LATS1 at the CLS site is reduced in cells with low upstream kinase activity. Endogenous LATS1 was immunoprecipitated from WT or *MST1/2*; *MAP4K1-7* (9KO) HEK293A cells and analyzed for phosphorylation at the CLS, AL, and HM sites. *B*–*D*, overexpression of upstream kinases enhances LATS2 CLS phosphorylation. In *LATS1/2* dKO HEK293A cells, HA-LATS2 and/or FLAG-tagged kinases MST1 (*B*), MST2 (*C*), and MAP4K4 (*D*) were overexpressed, and LATS2 phosphorylation at the CLS, AL, and HM sites was assessed by immunoblotting. *E*, CLS phosphorylation is enhanced in the active HM-D/E mutants of LATS2. FLAG-tagged WT and HM-D/E (T1041D/E) LATS2 were expressed in *LATS1/2* dKO HEK293A cells, immunoprecipitated, and analyzed by immunoblotting. *F*, CLS phosphorylation is abolished in the kinase-inactive LATS2 KR mutant. WT and KR (K697R) LATS2 were analyzed as above. *G*, LATS2 undergoes autophosphorylation at the CLS and AL sites *in vitro*. FLAG-LATS2 was immunoprecipitated and subjected to an kinase assay with or without ATP (500 μM). *H*, LATS2 CLS-A (S835A) and AL-A (S872A) mutants mutually reduce each other’s phosphorylation, indicating coordination between the CLS and AL sites. *I*, the CLS-A mutation attenuates AL phosphorylation in the active HM-D/E LATS2 mutants. *J*, the HM-D/E mutations promote CLS phosphorylation even in the absence of AL phosphorylation, indicating that CLS phosphorylation is not entirely dependent on the AL site.

Next, we investigated how the newly identified CLS sites of LATS kinases were phosphorylated. Previous work indicated that both MST1/2 and MAP4K1-7 cooperatively activate LATS1/2, and their simultaneous inactivation, such as in *MST1/2*; *MAP4K1-7* (9KO) cells, effectively suppressed LATS1/2 kinase activity (6, 41, 42). Consistent with this, LATS1 CLS phosphorylation was markedly reduced in *MST1/2*; *MAP4K1-7* (9KO) HEK293A cells (Fig. 2*A*). Conversely, overexpression of MST1, MST2, and MAP4K4 markedly increased LATS2 CLS phosphorylation (Fig. 2, *B*–*D*). Importantly, the changes in CLS phosphorylation closely paralleled those of AL and HM sites, indicating that phosphorylation of this newly identified site correlates with LATS kinase activity.

Given the high degree of homology between LATS1 and LATS2, we conducted subsequent investigations mainly on LATS2. To examine the phosphorylation level of CLS under LATS2 activation, we constructed LATS2 HM-D (T1041D, threonine-to-aspartate) and HM-E (T1041E, threonine-to-glutamate) mutants to mimic the activated LATS2 (43). The CLS phosphorylation was markedly increased in both active LATS2 mutants (Figs. 2*E* and S3*A*). In contrast, the kinase-dead LATS2 KR mutant, in which the ATP-binding site lysine 697 was mutated to arginine (44), showed no detectable phosphorylation at the CLS (Figs. 2*F* and S3*B*). The phosphorylation of the AL site was also induced in HM-D/E mutants and reduced in the KR mutant (Fig. 2, *E* and *F*). Given that CLS and AL phosphorylation exhibited similar patterns under LATS2 activation and inactivation, we hypothesized that CLS might also undergo autophosphorylation. Indeed, we purified LATS2 and performed an *in vitro* kinase assay; the phosphorylation at both CLS and AL sites increased gradually upon the addition of ATP (Fig. 2*G*).

To further investigate the interrelationship among the CLS, AL, and HM phosphorylation sites of LATS kinases, we analyzed LATS2 mutants with selective substitutions at each site. Mutation of either the CLS site (CLS-A) or the AL site (AL-A; S872A, serine-to-alanine) markedly reduced phosphorylation at AL and CLS, respectively, whereas HM phosphorylation was largely unaffected (Fig. 2*H*). Notably, in CLS-A and AL-A mutants, introducing an HM-D–activating mutation induced phosphorylation at the AL and CLS sites, respectively (Fig. 2, *I* and *J*). These results suggest that HM phosphorylation facilitates autophosphorylation at both CLS and AL sites, while CLS and AL can be phosphorylated independently, with their phosphorylation reciprocally reinforcing each other (Fig. S3*C*).

To confirm that LATS2 can autophosphorylate, we expressed FLAG-tagged LATS2-KR (a kinase-dead mutant) together with an EGFP-tagged LATS2 in *LATS1/2* double-knockout (dKO) cells. These two forms of LATS2 could be distinguished by their molecular weights. In cells expressing FLAG-LATS2-KR alone, the CLS site was not phosphorylated because LATS kinase activity was absent. When EGFP-LATS2 was co-expressed, phosphorylation at the CLS site of FLAG-LATS2-KR became detectable and was further enhanced upon co-expression of the active EGFP-LATS2-HM-D mutant (Fig. S3*D*). These results suggest that the CLS site of LATS2 can be regulated by intermolecular autophosphorylation (trans-autophosphorylation).

### CLS phosphorylation is required for the full activation of LATS2

**Figure 3:**
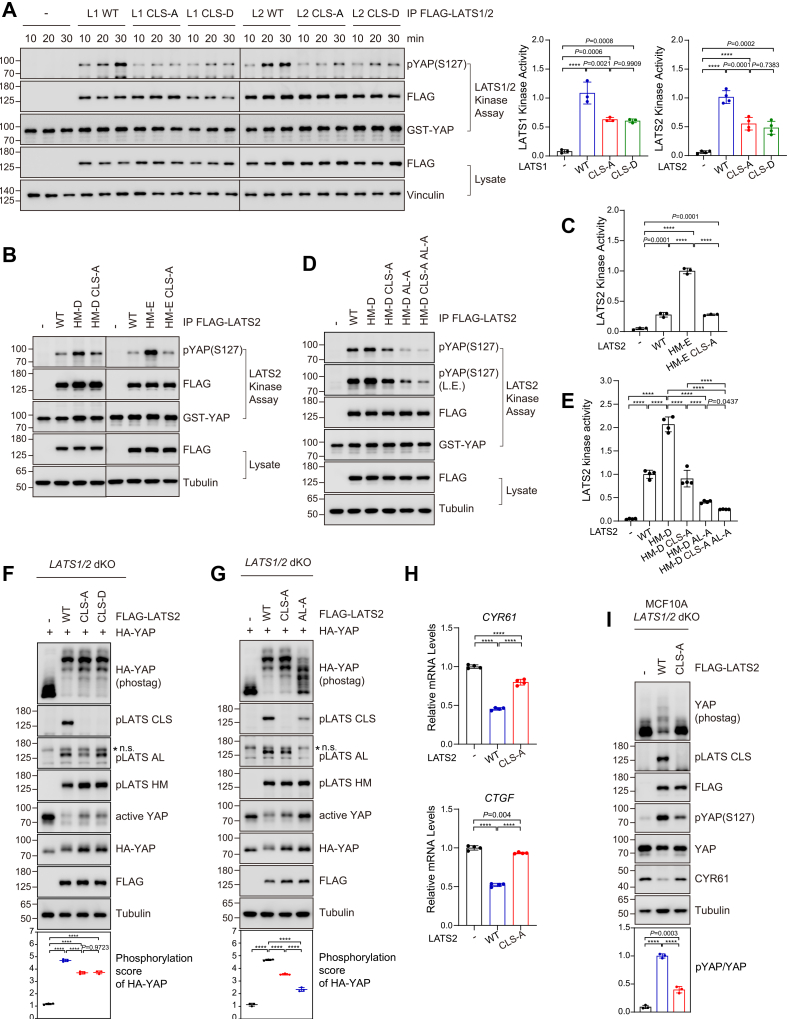
**Phosphorylation at the CLS site is essential for the full activation of LATS2.***A*, the CLS-A/D mutations markedly impair the kinase activity of LATS1/2. FLAG-tagged WT and mutants of LATS kinases were immunoprecipitated from *LATS1/2* dKO HEK293A cells and subjected to *in vitro* kinase assays using purified GST-YAP as substrate. L1 and L2 indicate LATS1 and LATS2, respectively. Quantification of kinase activity is shown on the *right*. *B*-*C*, introduction of the CLS-A mutation into the active HM-D/E mutants significantly reduces LATS2 kinase activity. FLAG-tagged WT and mutant LATS2 were immunoprecipitated from *LATS1/2* dKO HEK293A cells for an *in vitro* kinase assay. Quantification of kinase activity is shown in (*C*). *D*-*E*, combined AL-A and CLS-A mutations in the HM-D/E background cause a more pronounced reduction in kinase activity than either single mutation. FLAG-tagged WT and mutant LATS2 were immunoprecipitated from *LATS1/2* dKO HEK293A cells for an *in vitro* kinase assay. Quantification of kinase activity is shown in (*E*). *F*, CLS phosphorylation is critical for LATS2-dependent YAP phosphorylation. *LATS1/2* dKO HEK293A cells were cotransfected with HA-YAP and FLAG-LATS2 (WT or CLS mutants) and analyzed by immunoblotting. “n.s.” indicates a nonspecific signal. The phosphorylation levels of HA-YAP were quantified. See reference for details (69). *G*, the impact of the CLS-A mutation on YAP phosphorylation is weaker than that of the AL-A mutation. Cells were analyzed as in (*F*). *H*, the CLS-A mutation attenuates LATS2-mediated suppression of YAP target gene expression. WT or CLS-A LATS2 was reintroduced into *LATS1/2* dKO HEK293A cells, and mRNA expression levels of *CTGF* and *CYR61* were analyzed by qPCR. *I*, CLS phosphorylation is required for the full activation of LATS2 in *LATS1/2* dKO MCF10A cells. Cells reconstituted with WT or CLS-A LATS2 were analyzed by immunoblotting for YAP phosphorylation and *CYR61* expression. Ratios of pYAP/YAP are shown below. *p* values were assessed by one-way ANOVA with Tukey’s multiple comparisons test. Data are presented as mean ± S.D. from three independent biological replicates. *p* values are shown in the figure. ∗∗∗∗*p* < 0.0001.

We next examined whether CLS phosphorylation regulates LATS kinase activity. *In vitro* kinase assays showed that CLS-A and CLS-D (serine-to-aspartate) mutations markedly reduced LATS2 kinase activity. However, the reduction was less pronounced than that caused by AL-A and AL-D (serine-to-aspartate) mutations (Figs. 3*A* and S3*E*). Similar results were observed for the corresponding CLS mutants of LATS1 (Fig. 3*A*). Consistently, introduction of the CLS-A mutation into the active LATS2 mutants (HM-D/E) markedly attenuated their kinase activity (Fig. 3, *B* and *C*). The combined mutation of LATS2 AL-A and CLS-A caused a more pronounced reduction in kinase activity than the single mutant, and this inhibitory effect was more pronounced in the presence of active HM-D mutation (Fig. 3, *D*, *E* and S3, *F*–*H*). LATS1 and LATS2 directly phosphorylate and inactivate the downstream transcriptional co-factors YAP and TAZ (5, 45). We then investigated the effect of LATS2 CLS phosphorylation on YAP. When compared to WT LATS2, CLS mutants (CLS-A/D) showed a weaker effect on YAP phosphorylation, as indicated by the band shift in the presence of phos-tag and an increase in active YAP (dephosphorylated at S127) signals (Fig. 3*F*). LATS2 and its CLS mutants interacted with YAP at a comparable affinity; hence, the change in YAP phosphorylation was mainly due to reduced LATS2 kinase activity (Fig. S3*I*). Compared to the autophosphorylation AL site, the CLS-A mutation caused a weaker reduction in YAP phosphorylation (Fig. 3*G*). In contrast, a nearby phosphorylation site, tyrosine 824, when mutated to alanine (Y824A), showed no effect on YAP phosphorylation (Fig. S3*J*).

YAP induces a wide range of genes that regulate cell proliferation, differentiation, and survival (46, 47, 48). Among many target genes, cysteine-rich protein 61 (*CYR61*) and connective tissue growth factor (*CTGF*) are robustly induced by YAP across various cell types (49, 50). We then tested the effect of the CLS-A mutation on YAP transcriptional activity. Reintroduction of WT LATS2 into *LATS1/2* dKO HEK293A cells effectively repressed the expression of *CYR61* and *CTGF*, but the effect of the CLS-A mutant was much weaker (Fig. 3*H* and S3*K*). We also reintroduced WT LATS2 and the CLS-A mutant into *LATS1/2* dKO MCF10A cells and observed a similar effect on YAP phosphorylation and *CYR61* expression (Fig. 3*I*). In addition, we generated a *LATS1* knockout and *LATS2* CLS-A knock-in DLD1 cell line. The CLS-A knock-in markedly attenuated LATS2-mediated suppression of YAP (Fig. S3*L*). Collectively, these findings suggest that autophosphorylation of LATS2 at CLS is required for full LATS2 activation and subsequent inactivation of YAP-mediated transcription.

### CLS phosphorylation regulates YAP activity in cancer cells

**Figure 4:**
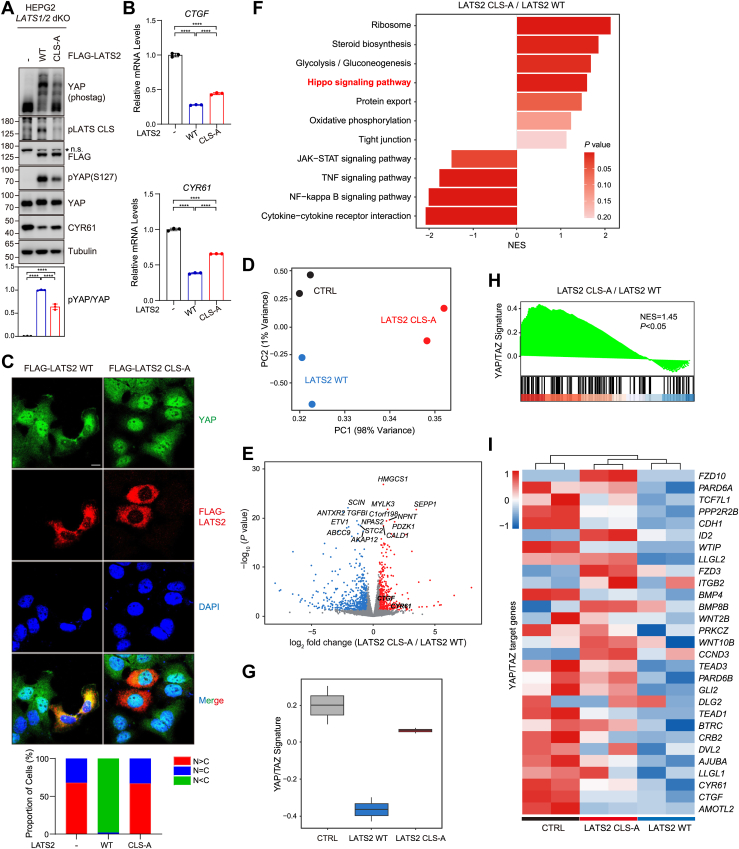
**LATS2 CLS phosphorylation modulates YAP activity in liver cancer cells.***A*, the CLS-A mutation in LATS2 attenuates YAP phosphorylation. FLAG-tagged WT or CLS-A LATS2 was reintroduced into *LATS1/2* dKO HEPG2 cells. YAP phosphorylation and the expression of *CYR61* were analyzed by immunoblotting. The ratios of pYAP/YAP were quantified. *B*, the CLS-A mutation compromises the ability of LATS2 to suppress YAP target gene expression in *LATS1/2* dKO HEPG2 cells. The mRNA expression levels of *CTGF* and *CYR61* were analyzed by qPCR. *C*, the CLS-A mutation in LATS2 impairs cytoplasmic retention of YAP. WT or CLS-A LATS2 was expressed in *LATS1/2* dKO HEPG2 cells, and subcellular localization of endogenous YAP was analyzed by immunofluorescence. Quantification was based on 100 cells per condition across three independent experiments. Merged images show YAP (*green*), FLAG-LATS2 (*red*), and nuclei (DAPI, *blue*). Scale bar represents 10 μm. *D*, principal component analysis (PCA) of RNA-seq data from HEPG2 cells expressing WT or CLS-A LATS2. *E*, volcano plot displaying differentially expressed genes in HEPG2 cells expressing WT or CLS-A LATS2, with genes of interest highlighted. *F*, Gene set enrichment analysis (GSEA) of differentially expressed genes in CLS-A *versus* WT LATS2-expressing cells. *G*-*I*, the CLS-A mutation weakens the ability of LATS2 to suppress YAP/TAZ-dependent transcription. YAP/TAZ signature scores (*G*), GSEA enrichment plot (*H*), and heatmap of YAP/TAZ target gene expression (*I*) are shown. *p* values were assessed by one-way ANOVA with Tukey’s multiple comparisons test. Data are presented as mean ± S.D. from three independent biological replicates. ∗∗∗∗*p* < 0.0001.

Dysregulation of the Hippo signaling pathway is frequently observed in cancers, including hepatocellular carcinoma and hepatoblastoma (51, 52, 53). The human cancer cell line HEPG2 is widely used as an *in vitro* model for liver cancer research (52, 54). We established *LATS1/2* dKO HEPG2 cells and stably expressed WT LATS2 or CLS-A mutant at comparable levels (Fig. 4*A*). Mutation of the CLS significantly weakened the inhibitory effect of LATS2 on YAP, as indicated by reduced YAP phosphorylation and elevated YAP target gene expression (Fig. 4, *A* and *B*). Phosphorylation of YAP prevents its nuclear localization and transcriptional co-activator function (55). In line with this mechanism, the CLS-A mutant failed to promote robust cytoplasmic retention of YAP as the WT LATS2 did (Fig. 4*C*). Thus, CLS phosphorylation of LATS2 is critical for the effective inhibition of YAP activity in HEPG2 liver cancer cells.

To further assess the global transcriptional impact of CLS phosphorylation, we performed RNA-seq using *LATS1/2* dKO HEPG2 cells as controls. Principal component analysis revealed distinct clustering of control, WT LATS2, and LATS2 CLS-A samples (Fig. 4*D*). Gene expression profiling followed by gene set enrichment analysis further showed that the CLS-A mutation attenuated the repression of Hippo pathway–related gene sets (Fig. 4, *E* and *F*). As expected, compared with WT LATS2, the CLS-A mutant displayed a weaker inhibitory effect on the expression of canonical YAP target genes (Fig. 4, *G*–*I*). Moreover, cells expressing CLS-A showed higher expression of genes associated with ribosomal function and metabolic pathways, including steroid biosynthesis and gluconeogenesis (Fig. 4*E*). As ribosomes are essential for protein synthesis and the regulation of cell proliferation, differentiation, and apoptosis, these results suggest a growth disadvantage of cells with LATS2 phosphorylation at CLS (56, 57).

To further validate the functional relevance of this site, we examined the kinase activity and YAP-suppressive capacity of LATS2 CLS-A, AL-A, and kinase-dead (KR) mutants expressed at comparable levels in *LATS1/2* dKO HEPG2 cells. The CLS-A mutation significantly impaired LATS2 kinase activity and its ability to suppress YAP. However, this effect was less pronounced than that of the AL-A or KR mutations (Fig. S4*A*). RNA-seq analysis revealed consistent trends (Fig. S4, *B*–*H*). These findings demonstrate that CLS phosphorylation contributes to LATS2-mediated YAP repression, albeit to a lesser extent than AL phosphorylation.

### Loss of CLS phosphorylation impairs the tumor-suppressive function of LATS2

**Figure 5:**
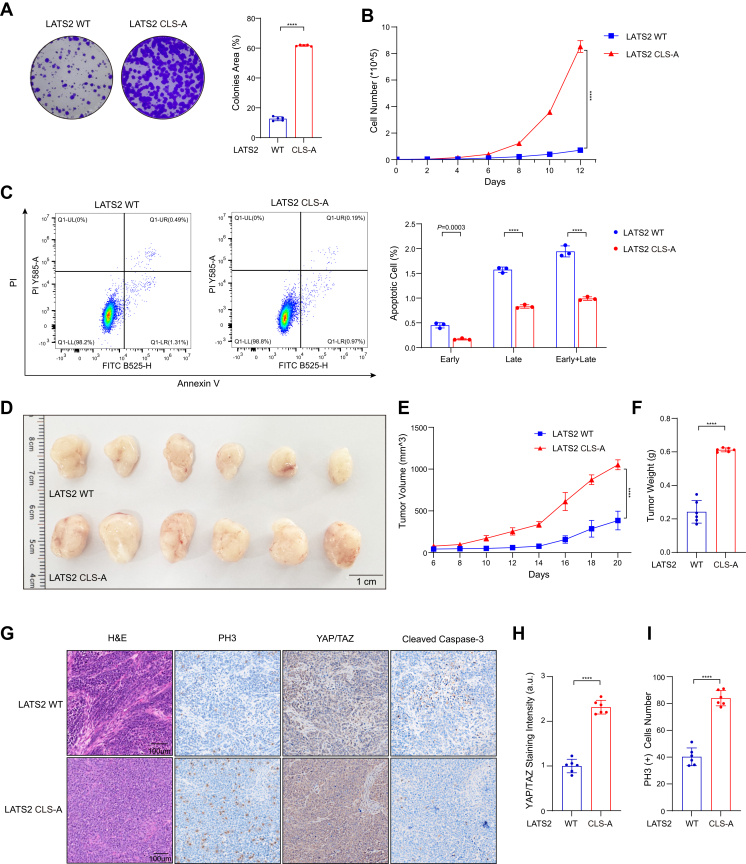
**CLS phosphorylation is required for LATS2 to suppress tumor growth.***A*, the CLS-A mutation compromises the ability of LATS2 to suppress colony formation in HEPG2 cells. Crystal violet staining was used to visualize and quantify colony-forming ability. Data are presented as mean ± S.D. from five independent biological replicates. *B*, the CLS-A mutation in LATS2 results in an impaired ability to suppress cell proliferation in HEPG2 cells. *C*, the CLS-A mutation impairs LATS2-induced apoptosis. Apoptotic cells were analyzed by Annexin V-FITC/PI staining followed by flow cytometry. Representative plots and quantification are shown. Early apoptotic cells were defined as Annexin V^+^/PI^-^, and late apoptotic cells as Annexin V^+^/PI^+^. *D*-*F*, the LATS2 CLS-A mutant exhibits reduced tumor-suppressive activity *in vivo*. Representative images of xenograft tumors derived from HEPG2 cells expressing WT or CLS-A LATS2; scale bar represents 1 cm (*D*). Tumor growth curves (*E*) and tumor weights (*F*) are shown. Data represent mean ± S.D. from six tumors. *G*-*I*, histological and immunohistochemical analyses of tumors formed by WT or CLS-A LATS2-expressing HEPG2 cells. H&E and IHC staining for PH3, YAP/TAZ, and Cleaved Caspase-3 (*G*). Quantification of YAP/TAZ staining intensity and PH3-positive cell counts (*H*-*I*). Each data point represents the mean from three fields of view per tumor. Scale bar represents 100 μm. Data are presented as mean ± S.D. from three independent biological replicates. ∗∗∗∗*p* < 0.0001. *p* values are shown in the figure. The Two-way ANOVA test is used for tumor growth and cell proliferation curves (*B*, *E*), and the unpaired Student’s *t* test is used for other data.

In the mammalian liver, the Hippo signaling pathway functions as a tumor suppressor. Liver-specific deletion of Hippo pathway genes, such as *Nf2*, *Sav1*, *Wwc1/2*, or *Mst1/2*, in mice causes hepatomegaly and promotes tumor development (58, 59, 60, 61, 62). Next, we aimed to investigate how LATS2 phosphorylation at the CLS site influences liver cancer development using established HEPG2 cell lines. Compared with WT LATS2, overexpression of the CLS-A mutant significantly enhanced the clonogenic capacity of HEPG2 cells (Fig. 5*A*). In line with these data, the proliferation of HEPG2 cells expressing the CLS-A mutant was also increased (Fig. 5*B*). Furthermore, HEPG2 cells expressing CLS-A exhibited reduced apoptosis, including both early and late apoptotic events (Fig. 5*C*).

Since the phosphorylation of LATS2 at the CLS affects YAP/TAZ activity and cell proliferation, it is likely to influence tumor growth capacity *in vivo*. We injected HEPG2 cells overexpressing either WT LATS2 or the CLS-A mutant into nude mice subcutaneously and monitored tumor growth. Indeed, HEPG2 cells expressing CLS-A showed faster tumor growth, as indicated by increased tumor volume and weight (Fig. 5, *D*–*F*). Immunohistochemical staining demonstrated that CLS-A tumors had higher expression of YAP/TAZ and Phospho-Histone H3 (PH3, a proliferation marker) and lower expression of Cleaved Caspase-3 (an apoptosis marker) (Fig. 5, *G*–*I*). Collectively, these results indicate that phosphorylation of LATS2 at the CLS site suppresses tumor formation in HEPG2 cells.

## Discussion

This study identifies CLS as a new autophosphorylation site within the kinase domain of LATS kinases. Biochemical and functional studies highlight a crucial role for CLS phosphorylation in activating LATS2 kinase and inhibiting YAP/TAZ transcriptional activity. Preventing phosphorylation at this site impairs the ability of LATS kinases to suppress cell growth and may contribute to tumor progression.

LATS kinases integrate diverse upstream signals to regulate the activity of the YAP/TAZ transcriptional coactivators, and thus their activity must be tightly controlled. LATS kinases are primed by phosphorylation at the HM site by upstream Hippo or Hippo-like kinases and subsequently activated by phosphorylation at the AL site. Here, we demonstrate that phosphorylation at the CLS site is also required for the full activation of LATS kinases. Both the CLS and AL sites are located within the unusually long activation loop around the catalytic center and are subject to autophosphorylation. Notably, structural studies of NDR/LATS family kinases suggest that the activation loop is highly flexible and conformationally heterogeneous, making this region difficult to model reliably using static structural models alone (63, 64). Although it remains unclear how these autophosphorylation events promote kinase activation, we speculate that the phosphorylation of CLS or AL affects the open-close conformational switch of the catalytic center, hence the efficiency of enzymatic activity. Moreover, CLS may act as a barrier to incoming substrates, and its phosphorylation may release it from the catalytic center, allowing YAP/TAZ and other substrates to be phosphorylated. A structural comparison between fully phosphorylated and dephosphorylated LATS kinases may provide valuable insights into these underlying molecular mechanisms. Meanwhile, similar to the AL site, both phospho-deficient and phospho-mimetic substitutions at the CLS site impaired LATS kinase activity. These observations suggest that static substitutions cannot fully recapitulate the dynamic regulatory effect of phosphorylation at this site. At the same time, we cannot completely exclude the possibility that CLS mutations may repress kinase activity by affecting local structure. This could be addressed by future structural studies, including analyses of mutant structures and molecular dynamics simulations.

Phosphorylation at the CLS site is sensitive to various upstream signals, such as serum availability and cell density, suggesting that it is a regulated phosphorylation event, consistent with previous reports showing that this site can be phosphorylated (Fig. 1) (65). We show that this site can undergo intermolecular autophosphorylation (Fig. S3*D*). However, whether intramolecular phosphorylation occurs *in vivo* remains to be determined. Future studies using single-molecule approaches may help resolve this question. Interestingly, a previous report demonstrated that CHK1 can phosphorylate the CLS site of LATS2 in response to UV irradiation (66). Therefore, CLS phosphorylation may be regulated through multiple mechanisms depending on the cellular microenvironment.

LATS kinases represent ideal molecular targets for cancer therapies and regenerative medicine. Inhibitors of LATS kinases have been developed to facilitate tissue repair and organ regeneration (67, 68). In contrast, activation of LATS kinases has been demonstrated to inhibit tumorigenesis (42, 69). Since regulation of CLS phosphorylation is critical for maintaining LATS kinase activity, it should be considered when designing new molecular probes targeting LATS kinases. The phosphorylation at this site may influence the affinity of potential inhibitors or activators for LATS kinases.

## Experimental procedures

### Plasmid construction and transfection

To generate expression plasmids, the target complementary DNA (cDNA) coding sequences were amplified by PCR using PrimeSTAR Max DNA Polymerase (Takara, #R045A). The PCR products were separated by agarose gel electrophoresis, purified using the DNA Gel Extraction Kit (Tsingke, #TSP602-200), and subsequently cloned into FLAG-tagged pLVX-puro or pLVX-hygro vectors (Takara, #632164) using the ClonExpress MultiS One Step Cloning Kit (Vazyme, #C113). The plasmid constructs were verified by restriction enzyme digestion and confirmed by Sanger sequencing. Plasmid DNA transfection was performed using PEI (1 mg/ml, pH 7.0) (Polysciences, #23966-2) or PolyJet (Signagen Laboratories, #SL100688) according to the manufacturer’s instructions.

### Lentiviral production and infection

For lentivirus production, HEK293T cells were cotransfected with pLVX-based plasmids and the packaging plasmids psPAX2 (Addgene, #12260) and pMD2.G (Addgene, #12259) at a ratio of 4:3:1 using PEI as the transfection reagent. Virus-containing medium was collected at 48 and 72 h post-transfection, filtered through a 0.45 μm filter (Millipore, #SLHP033RS), and centrifuged at 32,000 rpm for 2 h at 4 °C. Cells were infected with virus in the presence of 10 μg/ml polybrene (Sigma-Aldrich, #TR-1003-G), at 48 h after infection; stable cells were selected with 1 μg/ml puromycin (InvivoGen, #ant-pr-1) or 100 μg/ml hygromycin B (InvivoGen, #ant-hg-1).

### CRISPR/Cas9-mediated gene editing

To achieve gene knockout in specific cell lines, the CRISPR/Cas9 system was utilized. Gene-specific single-guide RNAs (sgRNAs) were designed using the CRISPR design tool (http://www.e-crisp.org/E-CRISP/) and cloned into the lentiCRISPR v2 vector (Addgene, #52961) using T4 DNA ligase (Takara, #2011A). All constructs were validated by Sanger sequencing. Cells transfected with Cas9 plasmids were selected with 1 μg/ml puromycin for 2 to 3 days. Monoclonal cell lines were established by limiting dilution into 96-well plates, and positive clones were identified through Western blot analysis or Sanger sequencing. The sgRNA sequences used for *LATS1* and *LATS2* knockout were 5′-CGACGAGAGCAGATGGCTGC-3′ and 5′-ACGTCAAGGCCGAGAGGGAC-3′, respectively. Using CRISPR/Cas9-mediated genome editing, *LATS2* CLS-A knock-in cells were generated in DLD1 *LATS1* knockout cells *via* electroporation (Fig. S5*E*). The donor fragment consisted of the LATS2 CLS-A sequence with 500 bp homology arms flanking the mutation. The sgRNA sequence used was 5′-CCATCCACGGCAAGATAGCA-3′.

### Cell lines and cell culture

Cells were maintained in tissue culture incubators at 37 °C with 5% CO_2_. HEPG2, HEK293A, and HEK293T cell lines were cultured in Dulbecco’s modified Eagle’s medium (DMEM) supplemented with 10% fetal bovine serum. The MCF10A cell line was cultured in DMEM/F12 supplemented with 5% horse serum, 20 ng/ml EGF, 1 mg/ml hydrocortisone, 100 ng/ml cholera toxin, and 10 μg/ml insulin. The culture medium was supplemented with 1% penicillin/streptomycin (Meilunbio, PWL062). In this study, *LATS1/2* knockout cell lines were generated in HEPG2 and MCF10A cells using the CRISPR/Cas9 system, with gene deletion confirmed *via* Western blotting and Sanger sequencing (Fig. S5, *A*–*D*). *LATS1/2* dKO and *MST1/2; MAP4K1-7* (9KO) HEK293A cell lines were described previously (6, 29). Stable cell lines expressing WT and mutant LATS2 were established through lentiviral transduction. HEK293T cells were obtained from the National Collection of Authenticated Cell Cultures (Cat# SCSP-502). DLD1 cells were generously provided by Yanhui Xu Lab. HEK293A cells were generously provided by Kun-Liang Guan Lab. HEPG2 and MCF10A cells were obtained from the Cell Bank of the Chinese Academy of Sciences.

### RNA interference

Cells were plated in 12-well plates with 500 μl of DMEM per well at a density of 30 to 50% and cultured for 12 h before transfection. For transfection, 6 pmol siRNA and 1 μl Lipofectamine RNAiMAX Transfection Reagent (Invitrogen, #13778075) were each diluted in 50 μl serum-free DMEM, mixed gently, and incubated at room temperature for 10 to 20 min. The transfection complex was added to the corresponding wells, and the plate was gently rocked to ensure even distribution. Cells were then incubated for 24 to 48 h prior to analysis. siRNAs were purchased from GenePharma. The detailed sequence information is as follows: si*STRIP1*: 5′-CAGCATCAAAGTGATTCGCAA-3′; si*STRIP2*: 5′-GCAGCCGAGTTGTCAGAAT-3′.

### Immunoblotting

Cells were directly lysed in 1 × SDS loading buffer (50 mM Tris–HCl at pH 6.8, 2% SDS, 10% glycerol, 0.025% bromophenol blue, and 1% β-mercaptoethanol) to obtain whole-cell lysates. Proteins were separated by SDS-PAGE and transferred onto nitrocellulose membranes. The membranes were blocked with 5% nonfat dry milk at room temperature for 1 h, then incubated overnight at 4 °C with primary antibodies diluted in 5% bovine serum albumin (BSA). After washing, the membranes were incubated with horseradish peroxidase (HRP)-conjugated secondary antibodies diluted in 5% nonfat dry milk for 1 h at room temperature. Chemiluminescent signals were detected using the High-sig ECL Immunoblotting Substrate (Tanon, #180-501) and captured using the 5200S imaging system (Tanon). Phospho-YAP protein was separated using phos-tag gels. Phos-tag gel was made by adding phos-tag reagents (10 μg/ml, FUJIFILM) and MnCl_2_ (50 μM) to a 7.5% SDS-PAGE gel according to the manufacturer’s instructions.

### Immunoprecipitation

Cultured cells were lysed in ice-cold RIPA buffer (10 mM Tris–HCl at pH 8.0, 140 mM NaCl, 1 mM EDTA, 0.5 mM EGTA, 1% Triton X-100, 0.1% sodium deoxycholate, and 0.1% SDS) or mild lysis buffer (50 mM Hepes at pH 7.5, 150 mM NaCl, 1 mM EDTA, 1% NP-40, 10 mM pyrophosphate, 10 mM glycerophosphate, 50 mM NaF, and 1.5 mM Na3VO4) supplemented with 1 mM PMSF, protease inhibitor cocktail (Selleckchem, #B14001), and phosphatase inhibitor cocktail (Selleckchem, #B15001). Whole-cell lysates were centrifuged at 12,000 rpm for 15 min at 4 °C, and the supernatants were used for immunoprecipitation. The indicated antibody was added to the supernatants and incubated for 1 h at 4 °C, followed by incubation with protein A agarose beads (Repligen, #10-1003-02) or protein A/G magnetic beads (Pierce, #88803) for an additional 2 h to capture the protein–antibody complex. Beads were washed four times with ice-cold RIPA buffer or mild lysis buffer. The immunoprecipitated proteins were dissolved in 1 × SDS-PAGE sample buffer and analyzed by immunoblotting.

### Immunofluorescence

Cells were cultured on fibronectin-coated coverslips, washed three times with PBS, fixed with 4% paraformaldehyde for 15 min, and permeabilized with 0.2% Triton X-100 for 10 min. After blocking with 3% BSA and 3% goat serum in PBS at room temperature for 1 h, the cells were incubated overnight at 4 °C with the specified primary antibody dilution. The cells were then washed three times and incubated with Alexa Fluor 488– and Alexa Fluor 555–conjugated secondary antibodies (Thermo Fisher Scientific) and DAPI (Sigma, #D9542) at room temperature for 1 h. Coverslides were then mounted, and images were captured and analyzed with a Zeiss LSM900 confocal microscope. A rabbit monoclonal anti-FLAG tag antibody (CST, #14793S,1:500 diluted) was used to detect FLAG-tagged LATS2 WT or CLS-A mutant. A mouse monoclonal anti-YAP1 antibody (Santa Cruz Biotechnology, #sc-101199, 1:100 diluted) was used to detect YAP.

### Immunohistochemistry

Tumor tissues were fixed in general-purpose tissue fixative solution (Servicebio, #G1101-500ML), embedded in paraffin, sectioned, and baked at 65 °C for 1 h. Following deparaffinization and rehydration, heat-mediated antigen retrieval was carried out in sodium citrate buffer (Beyotime, #P0083) at 100 °C for 15 min. Endogenous peroxidase activity was subsequently quenched using the Rabbit Two-step Method detection kit (Zsbio, #PV-9001). After blocking with 3% BSA in PBS with Tween 20 for 1 h at room temperature, the anti-Phospho-Histone H3 (Ser10) (CST, #9701, 1:200 diluted), anti-Cleaved Caspase-3 (Asp175) (CST, #9664, 1:200 diluted), and anti-YAP/TAZ (CST, #8418, 1:200 diluted) primary antibodies were incubated at 4 °C overnight. Subsequently, signal enhancer and HRP-conjugated goat anti-rabbit IgG polymer were sequentially applied according to the manufacturer’s instructions (Zsbio, #PV-9001). Visualization was achieved using DAB reagent (GeneTech, #GK500705). The sections were then counterstained with hematoxylin, mounted, and imaged using an Olympus VS200 Microscope.

### Dot blotting

Peptide samples were diluted in PBS to final concentrations of 1 μg/μl, 0.1 μg/μl, and 0.01 μg/μl. One microliter of each sample was spotted onto a nitrocellulose membrane and air-dried at room temperature for 30 min. The membrane was then blocked with 5% BSA for 1 h at room temperature, followed by incubation with the pCLS antibody (1:1000) at 4 °C overnight. After washing, the membrane was incubated with HRP-conjugated secondary antibody diluted in 5% BSA for 1 h at room temperature. Chemiluminescent signals were detected using the High-sig ECL Western blotting substrate and captured with a Tanon 5200S imaging system. Experiments were conducted in biological triplicates.

### Peptide competition assay

Proteins were separated by SDS-PAGE and transferred onto a nitrocellulose membrane. The membrane was blocked with 5% nonfat milk for 1 h at room temperature. The pCLS antibody was pre-incubated with different peptide samples (2 μg/ml) for 1 h at room temperature with gentle rotation and then incubated with the membrane at 4 °C overnight. Subsequent washing, incubation with HRP-conjugated secondary antibody, and signal detection were performed following Western blotting.

### RNA extraction, reverse transcription, and quantitative real-time PCR

Total RNA from adherent cells was extracted using the EZ-press RNA Purification Kit (EZB, #B0004DP) and then reverse-transcribed to cDNA using One-Step gDNA Removal and cDNA Synthesis SuperMix (TransGen, #AU311) following the manufacturer’s instructions. Quantitative PCR was conducted using TB Green Premix Ex Taq (TaKaRa, #RR420A) on the 7500 Real-Time PCR system (Applied Biosystems). All the quantitative real-time PCR experiments were conducted in biological triplicates. Primer sequences used in this study were as follows: *CYR61*, F:5′-AGCCTCGCATCCTATACAACC-3′, R:5′-TTCTTTCACAAGGCGGCACTC-3′; *CTGF*, F:5′-CCAATGACAACGCCTCCTG-3′, R:5′-TGGTGCAGCCAGAAAGCTC-3′; *GAPDH*, F:5′-ATGGGGAAGGTGAAGGTCG-3′, R:5′-GGGGTCATTGATGGCAACAATA-3′.

### Kinase assay

FLAG-tagged WT and LATS2 mutants were overexpressed in *LATS1/2* dKO HEK293A cells. Following lysis, cell lysates were subjected to immunoprecipitation using anti-FLAG affinity beads (Smart-Lifesciences, #SA042001). Proteins on beads were washed twice with the kinase assay buffer (30 mM Hepes, 50 mM potassium acetate, 5 mM MgCl_2_). GST-YAP was expressed and purified as previously reported (32). GST-YAP (0.5 mg/reaction) was used as the substrate for LATS kinases. The kinase reaction was performed at 30 °C for 30 min in the presence of 500 μM cold ATP, then terminated by the addition of SDS sample buffer, followed by immunoblotting. Phospho-specific antibody pYAP (S127) was used to evaluate the kinase activity of LATS1/2. In the λ-phosphatase assay, 200 U of phosphatase (Beyotime, #P2316S) was added to each reaction and incubated at 30 °C for 15 min according to the manufacturer’s instructions, followed by termination with SDS sample buffer. Phosphorylation of key sites in LATS2 was examined by immunoblotting.

### Colony formation assay

For the colony formation assay, cells (2 × 10^3^) were seeded into 6-well plates and cultured for 2 weeks. After incubation, cells were washed with PBS and fixed with 4% paraformaldehyde for 10 min. Colonies were then stained with 0.1% crystal violet (Servicebio, #G1014) at room temperature for 15 min. Excess dye was aspirated, and the wells were washed three times with PBS. Residual liquid was removed before imaging. The colony area was quantified using the ImageJ plugin “ColonyArea”, and the percentage of colony area was calculated. Statistical analysis was performed using GraphPad Prism 10.

### Cell proliferation assay

Cells (1 × 10^3^) were seeded into 12-well plates, and cell numbers were measured every 2 days using a cell counter (Bio-Rad). Statistical analysis was performed using GraphPad Prism 10.

### Tumor xenograft model

All mouse experiments were approved by the Animal Ethics Committee of Shanghai Medical College, Fudan University, and carried out following institutional guidelines. To establish xenograft tumors, cells were resuspended in PBS at a concentration of 1 × 10^7^ cells/ml. A total of 100 μl of the cell suspension was subcutaneously injected into the dorsal flank of BALB/c nude mice. Tumor growth was monitored and measured every 2 days. Tumor volume was calculated using the modified ellipsoid formula: V = 1/2 × (length × width^2^). Mice were sacrificed when the average tumor volume reached 1 cm^3^. Subcutaneous tumors were surgically removed, weighed, and imaged. The excised tissues were subsequently fixed in fixative solution and subjected to immunohistochemical analysis.

### Quantification and statistical analysis

Statistical analyses were performed using GraphPad Prism 10 software (GraphPad Software, Inc.). All representative experiments were repeated at least three times, as reported in the figures and corresponding legends. The results were expressed as the mean ± S.D. Statistical significance was determined using a one-way ANOVA test for most datasets. Student’s *t* test was used when two groups were compared. *p* values are shown in the figure. ∗∗∗∗*p* < 0.0001.

## Data availability

The RNA-seq data generated in this study have been deposited in the Genome Sequence Archive (GSA) at the National Genomics Data Center, China National Center for Bioinformation/Beijing Institute of Genomics, Chinese Academy of Sciences, under accession number HRA017418, and are publicly accessible at https://ngdc.cncb.ac.cn/gsa. The mass spectrometry proteomics data have been deposited to the ProteomeXchange Consortium *via* the PRIDE partner repository with the dataset identifier PXD076693 (70).

## Supporting information

This article contains supporting information.

## Conflicts of interest

The authors declare that they have no conflicts of interest with the contents of this article.
