# Supplementary material for: LATS kinase activity and tumor suppressor function are regulated by a second autophosphorylation site
Source: J Biol Chem. 2026 May 20;302(7):113177. doi: 10.1016/j.jbc.2026.113177 (PMC13292594; doi:10.1016/j.jbc.2026.113177)
Supplement: Supplementary Data [file mmc1.docx]

**SUPPLEMENTARY FIGURES AND FIGURE LEGENDS**

**
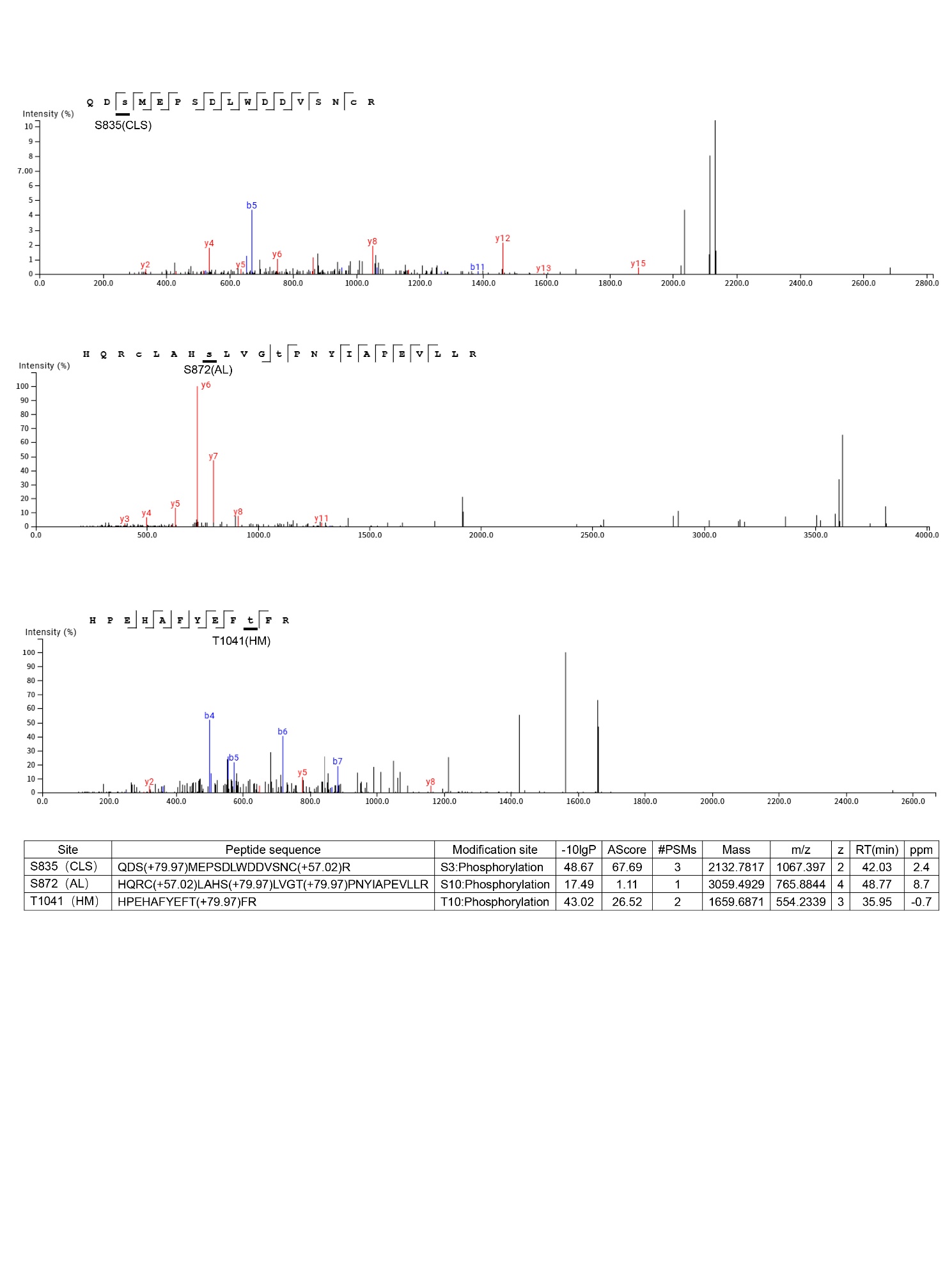
**

**Figure S1. Identification of the CLS phosphorylation site in LATS kinases.**

Phosphorylation of S835 (CLS), S872 (AL), and T1041 (HM) of LATS2 was identified by mass spectrometry. Human LATS2 was purified from HEK293A cells for mass spectrometry analysis.


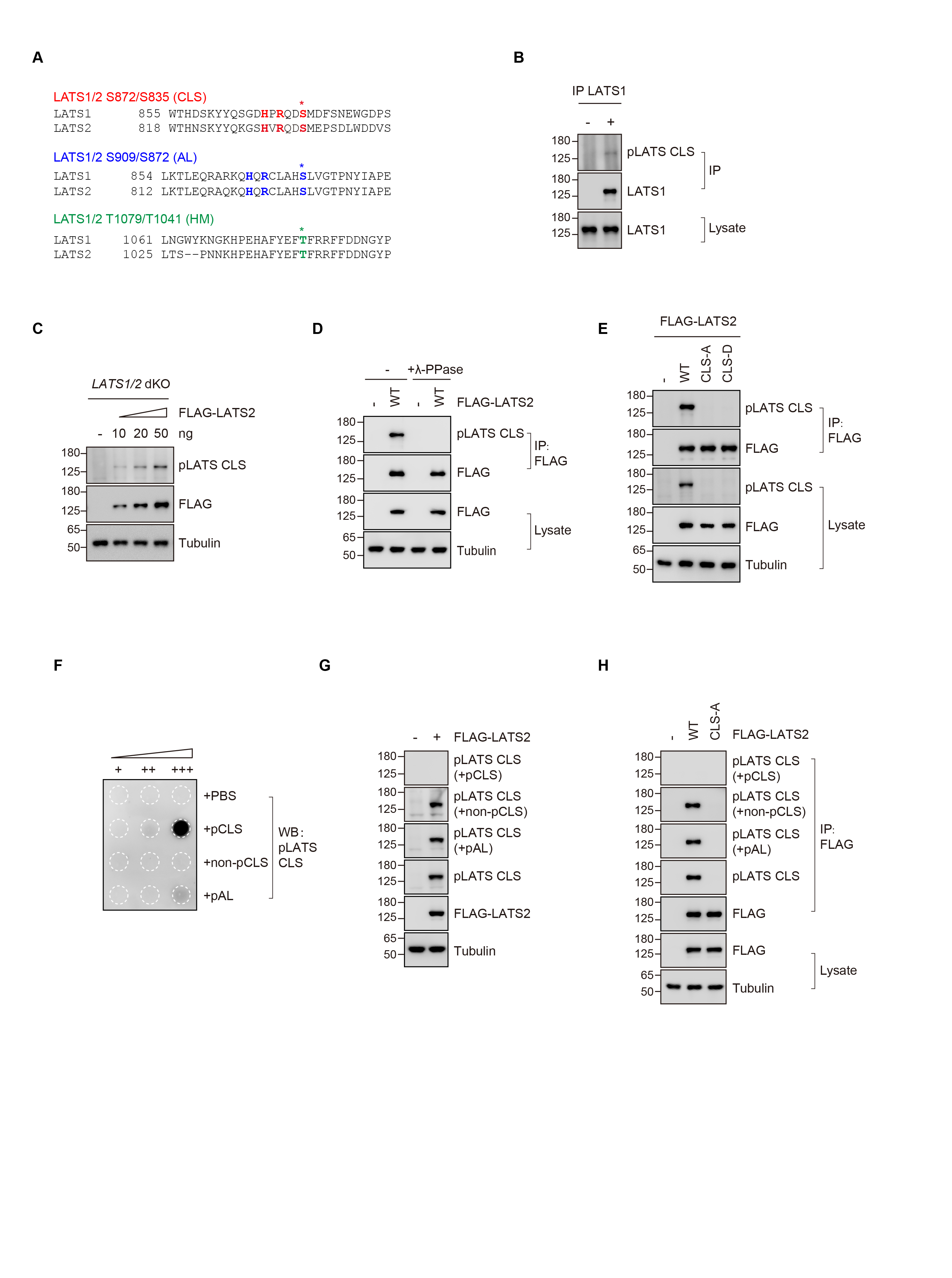


**Figure S2. Generation and validation of a phospho-specific pCLS antibody.**

(A) Alignment of the sequences surrounding the CLS, AL and HM sites in the human LATS1 and LATS2.

(B-C) Validation of the phospho-specific antibody against the CLS site in LATS kinases. The pCLS antibody effectively detects phosphorylation at S872 in immunoprecipitated LATS1 (B), and at S835 in ectopically expressed LATS2 (C).

(D) Treatment with λ-phosphatase abolishes pCLS antibody recognition of LATS2. λ-phosphatase (200 U) treatment confirms that the observed pCLS signal represents a genuine phosphorylation event.

(E) The pCLS antibody does not recognize the CLS mutants of LATS2. FLAG-tagged WT or CLS-A/D LATS2 was immunoprecipitated and analyzed by immunoblotting.

(F) The pCLS antibody shows no cross-reactivity with the AL site of LATS2. In dot blotting, the pCLS antibody was incubated with phosphorylated CLS (pCLS), non-phosphorylated CLS (non-pCLS), and phosphorylated AL (pAL) peptides on a nitrocellulose membrane, and signal intensity was analyzed by immunoblotting. The dashed circle indicates the position of the sample.

(G-H) The phosphorylated CLS peptide effectively blocks the recognition of LATS2 by the pCLS antibody. In peptide competition assay, the pCLS antibody was preincubated with pCLS, non-pCLS, or pAL peptides, and then used for immunoblotting of immunoprecipitated wild-type LATS2 and mutants.


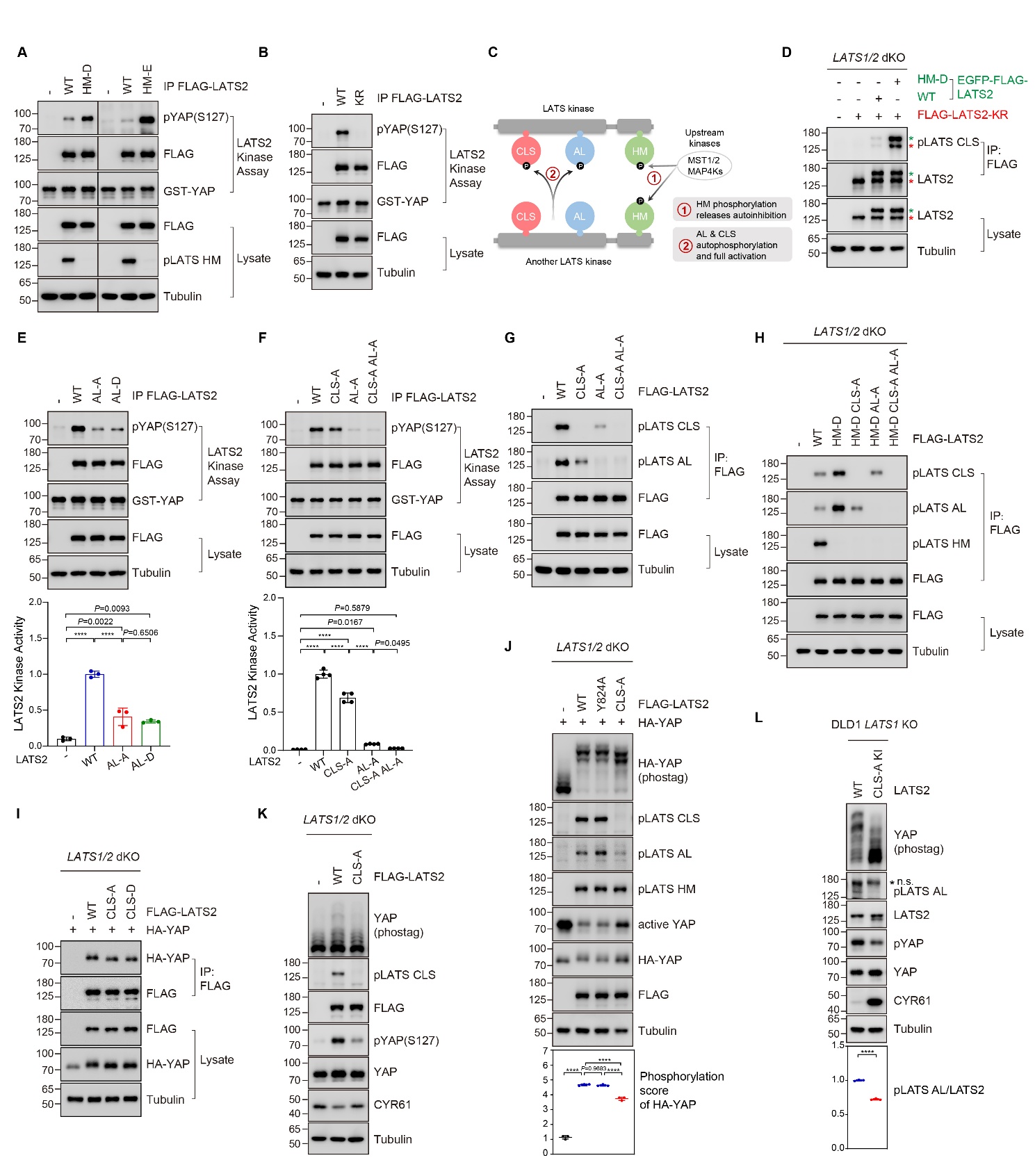


**Figure S3. Phosphorylation at the CLS site of LATS2 modulates its kinase activity and downstream YAP regulation.**

1. Phospho-mimetic HM-D/E mutations markedly enhance LATS2 kinase activity. FLAG-tagged WT and mutant LATS2 were expressed in *LATS1/2* dKO HEK293A cells, immunoprecipitated, and analyzed by an *in vitro* kinase assay.
2. The kinase-inactive KR mutation abolishes LATS2 kinase activity. WT and KR mutant LATS2 were analyzed as in (A).
3. Schematic model illustrating the interplay among CLS, AL, and HM phosphorylation in regulating LATS kinase activity.
4. The CLS site can be modified by trans-autophosphorylation. FLAG-LATS2-KR was expressed alone or co-expressed with EGFP-FLAG-LATS2 WT or HM-D mutant in *LATS1/2* dKO HEK293A cells. Lysates were subjected to immunoprecipitation using anti-FLAG antibody. FLAG-LATS2-KR and EGFP-FLAG-LATS2 were distinguished by molecular weight (*red* and *green*, respectively).
5. The AL-A/D mutations significantly reduce LATS2 kinase activity. FLAG-tagged WT and mutant LATS2 were expressed in *LATS1/2* dKO HEK293A cells, immunoprecipitated, and analyzed by an *in vitro* kinase assay.
6. Combined AL-A and CLS-A mutations cause a greater reduction in kinase activity than either single mutation. FLAG-tagged WT and mutant LATS2 were expressed in *LATS1/2* dKO HEK293A cells, immunoprecipitated, and analyzed by an *in vitro* kinase assay.

(G-H) Analysis of phosphorylation at key sites in the LATS2 CLS-A and AL-A double mutants. The precipitated proteins were analyzed by immunoblotting.

1. Wild-type LATS2 and CLS mutants display comparable binding to YAP. FLAG-tagged WT and CLS mutant LATS2 were co-expressed with HA-YAP in *LATS1/2* dKO HEK293A cells. Lysates were subjected to immunoprecipitation using anti-FLAG antibody. The precipitated proteins and whole cell lysates were analyzed by immunoblotting.
2. The Y824A mutation in LATS2 does not affect its regulation of YAP. FLAG-tagged WT and Y824A LATS2 were co-expressed with HA-YAP. HA-YAP phosphorylation levels were quantified.
3. The CLS-A mutation weakens the ability of LATS2 to suppress endogenous YAP activity. *LATS1/2* dKO HEK293A cells were reconstituted with FLAG-tagged WT or CLS-A LATS2, and endogenous YAP phosphorylation and *CYR61* expression were analyzed by immunoblotting.
4. *LATS2* CLS-A knock-in (KI) significantly weakens LATS2-mediated YAP suppression. *LATS2* CLS-A KI was performed in DLD1 *LATS1* KO cells, and endogenous YAP phosphorylation and *CYR61* expression were analyzed by immunoblotting.

*p* values were assessed by one-way ANOVA with Tukey’s multiple comparisons test. Data are presented as mean ± S.D. from three independent biological replicates. *p* values are shown in the figure. *****p* < 0.0001.


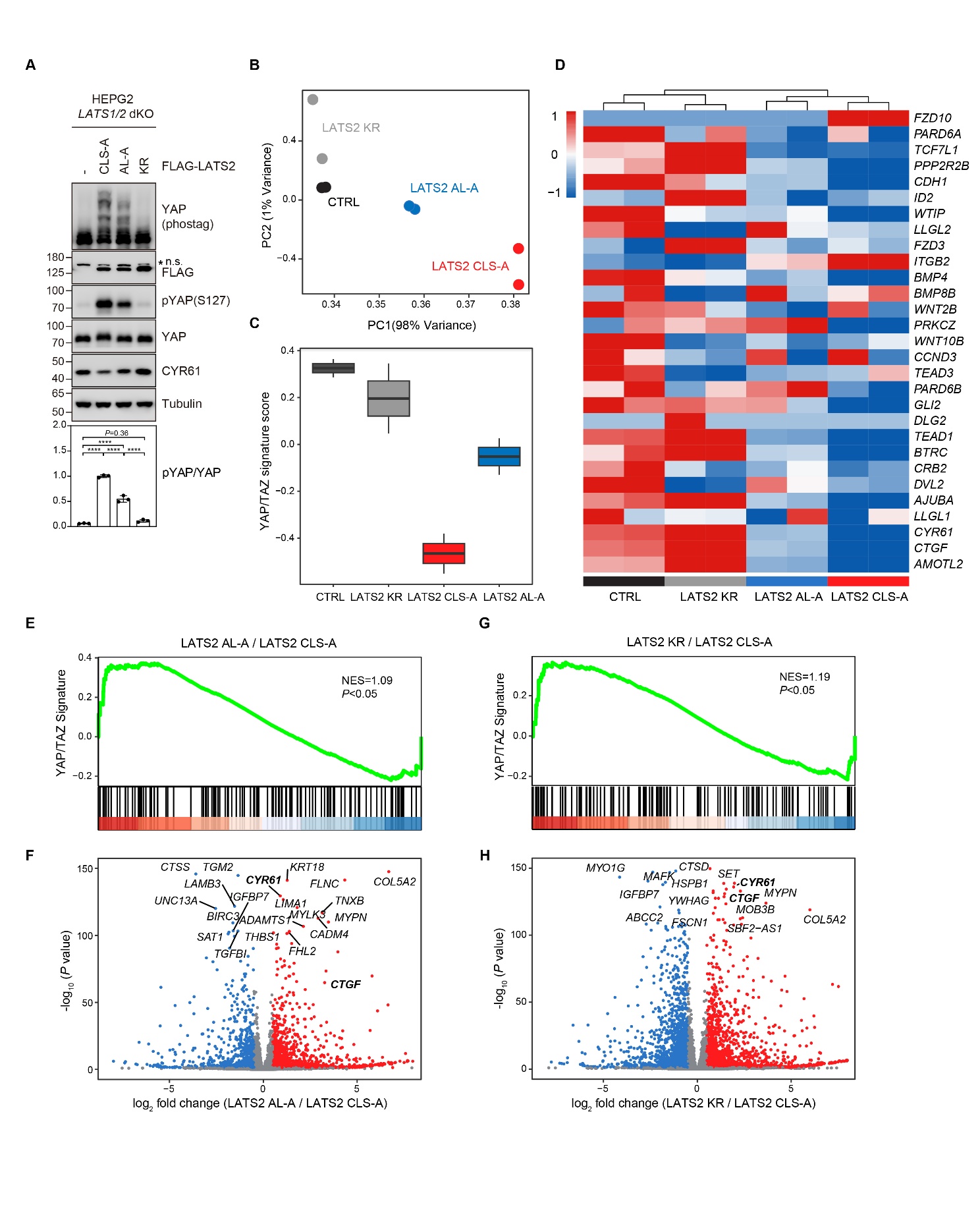


**Figure S4. CLS and AL phosphorylation of LATS2 differentially modulate YAP activity in liver cancer cells.**

1. Comparison of the effects of LATS2 CLS-A, AL-A, and KR mutants on YAP phosphorylation. The CLS-A, AL-A, and KR mutants of LATS2 were reintroduced into *LATS1/2* dKO HEPG2 cells. YAP phosphorylation and the expression of *CYR61* were analyzed by immunoblotting. The ratios of pYAP/YAP were quantified.
2. Principal component analysis (PCA) was performed on *LATS1/2* dKO HEPG2 cells expressing LATS2 CLS-A, AL-A, or KR mutants.

(C-H) The LATS2 CLS-A, AL-A, and KR mutations attenuate its repression of YAP target gene expression. The YAP/TAZ signature score (C) and heatmap of differentially expressed genes (D) are shown. Gene set enrichment analyses (E and G) and volcano plots (F and H) show differentially expressed genes in *LATS1/2* dKO HEPG2 cells expressing LATS2 AL-A or KR mutants relative to CLS-A, with genes of interest highlighted.

*p* values were assessed by one-way ANOVA with Tukey’s multiple comparisons test. Data are presented as mean ± S.D. from three independent biological replicates. *p* values are shown in the figure. *****p* < 0.0001.


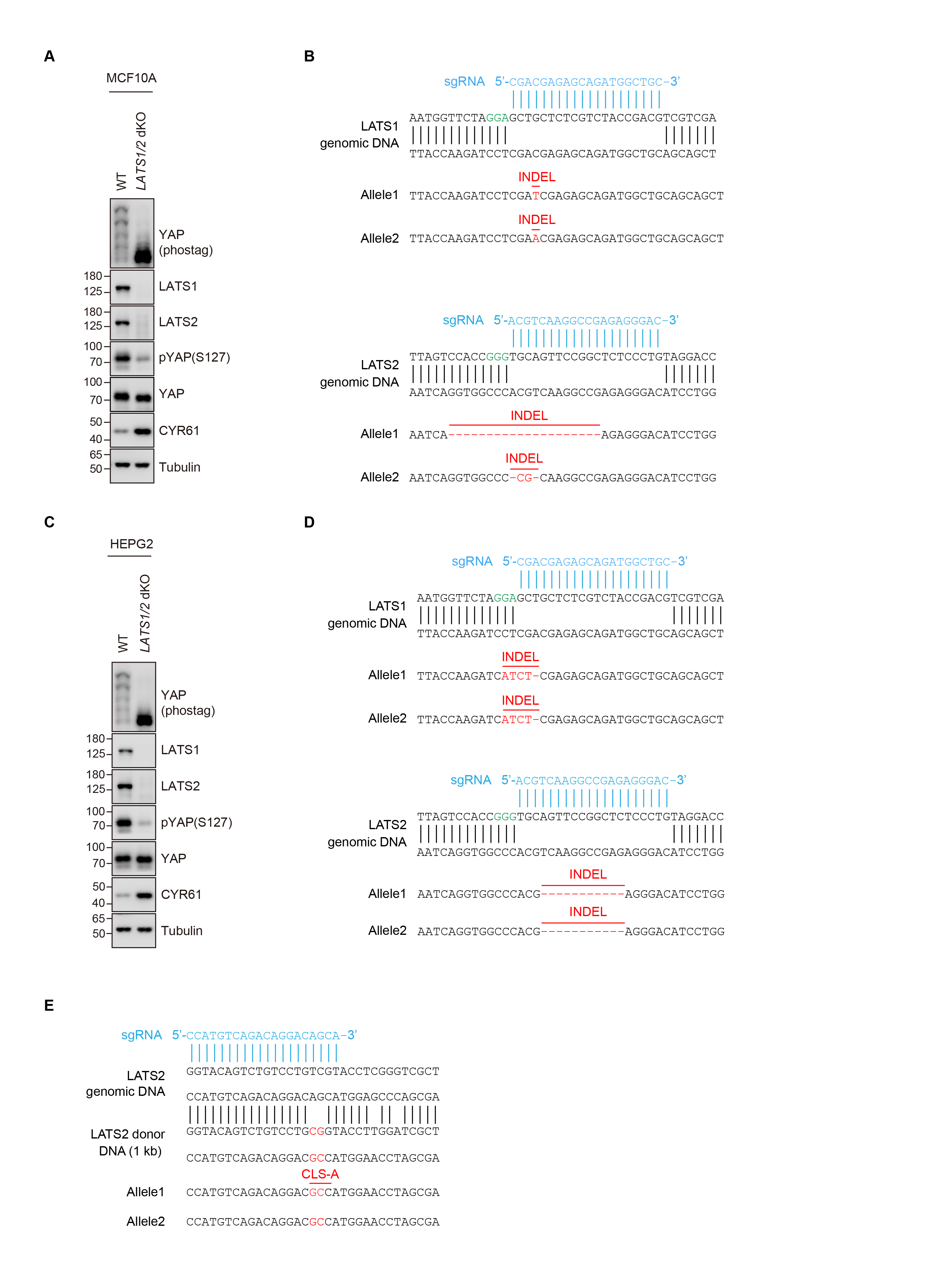


**Figure S5. Validation of knockout cell lines.**

(A-B) Immunoblotting and Sanger sequencing analysis of *LATS1/2* dKO in MCF10A cells.

(C-D) Immunoblotting and Sanger sequencing analysis of *LATS1/2* dKO in HEPG2 cells.

(E) Sanger sequencing analysis of *LATS2* CLS-A KI in DLD1 *LATS1* KO cells.

**Table S1. Information on all antibodies used in this study**

| **Antibodies** | **SOURCE** | **IDENTIFIER** |
| --- | --- | --- |
| Rabbit monoclonal anti-LATS1 (C66B5) | Cell Signaling Technology | Cat# 3477;  RRID: AB_2133513 |
| Rabbit monoclonal anti-LATS2 (D83D6) | Cell Signaling Technology | Cat# 5888;  RRID: AB_10835233 |
| Rabbit monoclonal anti-Phospho-LATS1 (Thr1079) (D57D3) | Cell Signaling Technology | Cat# 8654;  RRID: AB_10971635 |
| Rabbit monoclonal anti-Phospho-LATS1 (Ser909) | Cell Signaling Technology | Cat# 9157;  RRID: AB_2133515 |
| Rabbit monoclonal anti-HA Tag (HRP Conjugate) | Cell Signaling Technology | Cat# 14031;  RRID: AB_2798368 |
| Rabbit monoclonal anti-Non-phospho (Active) YAP (Ser127) (E6U8Z) | Cell Signaling Technology | Cat# 29495;  RRID: AB_2798974 |
| Rabbit monoclonal anti-YAP (D8H1X) | Cell Signaling Technology | Cat# 14074;  RRID: AB_2650491 |
| Rabbit polyclonal anti-Phospho-YAP (Ser127) | Cell Signaling Technology | Cat# 4911;  RRID: AB_ 2218913 |
| Rabbit monoclonal anti-Vinculin (E1E9V) | Cell Signaling Technology | Cat# 13901;  RRID: AB_2728768 |
| Normal Rabbit IgG | Cell Signaling Technology | Cat# 2729;  RRID: AB_1031062 |
| Rabbit monoclonal anti-YAP/TAZ (D24E4) | Cell Signaling Technology | Cat# 8418;  RRID: AB_10950494 |
| Rabbit monoclonal anti-DYKDDDDK Tag (D6W5B) | Cell Signaling Technology | Cat# 14793;  RRID: AB_2572291 |
| Rabbit monoclonal anti- Phospho-Histone H3 (Ser10) | Cell Signaling Technology | Cat# 9701;  RRID: AB_331535 |
| Rabbit monoclonal anti-Cleaved Caspase-3 (Asp175) (5A1E) | Cell Signaling Technology | Cat# 9664;  RRID: AB_2070042 |
| Mouse monoclonal anti-YAP1 (EP1674Y) | Santa Cruz Biotechnology | Cat# sc-101199;  RRID: AB_1131430 |
| Rabbit polyclonal anti-Cyr61 (H-78) | Santa Cruz Biotechnology | Cat# sc-13100;  RRID: AB_2088733 |
| Rabbit polyclonal anti-STRIP1 | Bethyl | Cat# A304-644A;  RRID: AB_2620839 |
| Rabbit polyclonal anti-STRIP2 | Sigma-Aldrich | Cat# HPA019657;  RRID: AB_1848399 |
| Mouse monoclonal anti-α-Tubulin | Sigma-Aldrich | Cat# T6199;  RRID: AB_477583 |
| Mouse monoclonal anti-Flag Tag (HRP conjugated) | Sigma-Aldrich | Cat# A8592;  RRID: AB_439702 |
| Goat anti-rabbit IgG antibody,Alexa Fluor 488 | Thermo Fisher Scientific | Cat# A11008;  RRID: AB_143165 |
| Goat anti-mouse IgG antibody,Alexa Fluor 555 | Thermo Fisher Scientific | Cat# A21422;  RRID: AB_2535844 |
| Peroxidase AffiniPure Goat Anti-Mouse IgG (H+L) | Jackson Immuno Research Laboratories | Cat# 115-035-003;  RRID: AB_10015289 |
| Peroxidase AffiniPure Goat Anti-Rabbit IgG (H+L) | Jackson Immuno Research Laboratories | Cat# 111-035-003;  RRID: AB_2313567 |
